# Bevacizumab for Glioblastoma—A Promising Drug or Not?

**DOI:** 10.3390/cancers5041456

**Published:** 2013-11-07

**Authors:** Motoo Nagane, Ryo Nishikawa

**Affiliations:** 1 Department of Neurosurgery, Kyorin University Faculty of Medicine, 6-20-2 Shinkawa, Mitaka-shi, Tokyo 181-8611, Japan; E-Mail: nagane-nsu@umin.ac.jp; 2 Department of Neuro-Oncology/Neurosurgery, International Medical Center, Saitama Medical University, 1397-1 Yamane, Hidaka-shi, Saitama-ken 350-1298, Japan

**Keywords:** bevacizumab, glioblastoma, chemotherapy

## Abstract

Two double blind, placebo-controlled, and randomized phase III studies were conducted, and the results including OS’s were reported at the ASCO Meeting in June 2013, which was the beginning of confusion surrounding this topic. This is a review article not only summarizing the previous evidence, but also looking beyond.

## 1. Introduction

Studies have confirmed angiogenesis as a complex and dynamic process occurring during the growth of all solid tumors beyond 2–3 mm in size and that tumors are angiogenesis dependent [1]. Vascular endothelial growth factor (VEGF) is one of the most important factors regulating angiogenesis in the most aggressive malignant brain tumor, glioblastoma (GBM) [2]. Bevacizumab is a humanized form of a mouse monoclonal antibody against human VEGF, which binds to and neutralizes mainly VEGF-A. The interaction between GBM stem cells located in their perivascular niche and endothelial cells may also be disrupted by anti-VEGF agents such as bevacizumab, contributing to eventual cell death [3].

Bevacizumab was first reported as a treatment for recurrent GBMs in 2005 [4]. The results showed significant improvement in radiological scans as well as in the patients’ symptoms and neurological function. In a retrospective analysis of 29 patients treated with a combination of bevacizumab and irinotecan, a topoisomerase-1 inhibitor, an overall response rate of 43% was observed, with one complete response (CR), eight partial responses (PR), and eleven patients with stable disease (SD). There was one death that occurred secondary to intracranial hemorrhage and one due to gastrointestinal perforation [4]. This initial efficacy of bevacizumab led to a phase II trial by Vredenburgh *et al*. in a total of 35 patients with recurrent GBMs, using also bevacizumab and irinotecan [5]. The overall results of this trial included a PFS at 6 months (PFS-6m) of 46% (95% confidence interval [CI]; 32%–66%). A PR was observed in 20 of 35 patients (57%. 95% CI; 39%–74%), with the median OS in the study being 10.5 months. A historical comparator of PFS-6m for recurrent GBM should be the data of temozolomide by Yung *et al*. that was 18% [6]. The adverse events in this bevacizumab study included one intracranial hemorrhage and four patients who developed thromboembolic events. Thus, bevacizumab was seen as a promising new therapeutic for treating patients with gliomas. A subsequent study conducted by Friedman *et al*., an open-label randomized phase II study, evaluated bevacizumab with and without irinotecan in 167 recurrent GBM patients (BRAIN study) [7]. A response rate of 37.8% and 28.2% in the bevacizumab plus irinotecan (*n* = 82) and the bevacizumab alone (*n* = 85) groups, respectively, and PFS-6m of 50.3% and 42.6%, respectively, was seen in this study.

## 2. Bevacizumab as a Single Agent *vs*. Combination Therapy for Recurrent GBM

One of the practical issues in treating recurrent GBM with bevacizumab is that combination with other chemotherapeutic agents has not been proved to increase efficacy of bevacizumab monotherapy. This is distinct from other malignancies where bevacizumab is approved and used in combination with cytotoxic agents, due to the fact no appreciable benefit is observed by using bevacizumab alone. For recurrent GBM, similar rates of response (20%–40%) and PFS-6m (23%–50%) were reported for bevacizumab plus chemotherapy (including irinotecan, carboplatin, nitrosoureas such as carmustine [1,3-Bis(2-chloroethyl)-1-nitrosourea; BCNU], lomustine[1-(2-chloroethyl)-3-cyclohexyl-1-nitrosourea; CCNU] and fotemustine, temozolomide (TMZ), erlotinib, etoposide, and enzastaurin) relative to bevacizumab monotherapy [7,8,9,10,11,12], having argued that it is unclear whether additional agents contribute to enhance the benefit of bevacizumab in the treatment of recurrent GBM. Among those, irinotecan showed a slight increase in efficacy, albeit with limited single-agent activity in the recurrent setting [7,13,14,15,16,17].

The lack of significant enhancement of efficacy by bevacizumab combinations might be partially attributable to the brain-specific microenvironment constituted with the blood-brain barrier (BBB), as compared to other organs. One of the hypothetical mechanisms for therapeutic effects by bevacizumab is normalization of VEGF-induced dysregulated vascular structure causing excessive increase in fluid leakiness from vessels, resulting in high interstitial pressure and low perfusion in the tumor tissue, thereby hampering sufficient drug delivery. A consequence of bevacizumab action is neutralization of vascular permeabilizing effects of VEGF and reducing interstitial edema, thus it is postulated that perfusion within tumor tissue is normalized, leading to improved drug delivery to tumor cells. However, in the brain, this process may be accompanied with repair of disrupted BBB function as well, which in turn would restrict penetration of most chemotherapeutic agents into the brain parenchyma [18,19]. One example was shown in a mouse xenograft model where TMZ was effective in inducing apoptosis and eradication of tumors derived from human glioma U87 cells, but the addition of a multitargeting anti-angiogenic inhibitor vandetanib to TMZ conversely resulted in a decrease in apoptosis rate and tumor suppression compared to TMZ monotherapy [20].

To explore this issue directly, randomized comparator trials of bevacizumab have been initiated in patients with recurrent GBM by a Dutch study (BELOB) and European Organisation for Research and Treatment of Cancer (EORTC; EORTC 26101). Both studies use lomustine (CCNU) as the standard comparator to bevacizumab, either as a single agent or in combination with bevacizumab, to evaluate whether bevacizumab alone is superior in improving survival to lomustine that has served as the standard therapy at progression in Europe, and whether the combination has a higher activity than bevacizumab monotherapy as well. The phase II three-arm Dutch study BELOB has recently closed with 140 patients randomly allocated to either bevacizumab alone, lomustine alone, or combination of these two agents using the RANO criteria for response assay. The primary endpoint, OS at 9 months, was 38%, 43%, and 59%, and PFS-6m was 16%, 13%, and 41%, respectively, demonstrating better activity for bevacizumab/lomustine combination therapy [21]. While PFS-6m in the bevacizumab/lomustine arm (41%) was consistent with other bevacizumab combination studies, considerably lower PFS-6m in the bevacizumab single arm (16%) in this study than in historical data (25%–43%) appears to correlate with the number of cycles administered before progression determined by Response Assessment in Neuro-Oncology (RANO) criteria. Here, the median number of cycles was two, with 28% of patients in the bevacizumab arm deemed PD after the first cycle. This data contrasted strikingly with the previous phase II studies of bevacizumab as a single agent where the median number of bevacizumab doses was nine (BRAIN trial, NCT00345163) and six (JO22506) [7,22]. Whether this difference comes from different methods in measuring progression or other more complex reasons, remains to be elucidated. 

An Australian randomized phase II trial (CABARET) compared bevacizumab with or without carboplatin to determine whether addition of another commonly used cytotoxic agent to bevacizumab may benefit survival in 122 patients with recurrent GBM. PFS-6m was 26% (combination) and 24% (bevacizumab monotherapy), and mOS was 6.9 months and 6.4 months, respectively, failing to result in clinical benefit with the combination [23].

A German randomized phase II trial (GLARIUS, NCT00967330) aimed to improve survival of patients with newly-diagnosed GBM having unfavorable unmethylated MGMT promoter, by treating with bevacizumab and radiotherapy followed by combination of bevacizumab plus irinotecan instead of TMZ. The primary endpoint of PFS-6m was 79.6% in the bevacizumab/irinotecan arm and 41.3% in TMZ arm (*p* < 0.0001). mPFS was 9.7 months (bevacizumab/irinotecan) *vs*. 6.0 months (TMZ), and mOS was 16.6 months (bevacizumab/irinotecan) *vs*. 14.8 months (TMZ). Among patients in the TMZ arm who had progressed or died (85%), the crossover rate (second line with bevacizumab/irinotecan) was 63% [24]. These results suggest that substitution of TMZ with irinotecan in combination with upfront bevacizumab use may benefit the survival of patients with MGMT-unmethylated newly diagnosed GBM, in contrast to the Radiation Therapy Oncology Group (RTOG) 0825 study (see below) where addition of bevacizumab to TMZ showed no survival benefits in this unfavorable prognostic population. These studies warrant further phase III trials investigating bevacizumab/irinotecan combination in the patients with MGMT-unmethylated newly diagnosed GBM.

## 3. Controversies

Following the results of the various trials in recurrent GBMs, there has been considerable interest in evaluating the benefit of bevacizumab in untreated newly diagnosed GBMs. There are two randomized phase III trials evaluating the role of bevacizumab in combination with TMZ and radiation therapy for newly diagnosed GBMs. RTOG 0825 is a large phase III trial targeting 720 patients and the other trial is the Effectiveness of Avastin in GBM (AVAglio) study targeting over 900 patients and sponsored by Hoffman-LaRoche (Basel, Switzerland). In November 2012 at the Society of Neuro-Oncology Meeting in Washington, D.C., Chinot *et al*. presented the interim results of the AVAglio study that included final PFS data and interim OS data. The result was positive for an improved PFS, with other promising outcomes in secondary endpoints such as quality of life (QoL). Specifically, the Investigator-Assessed PFS, that was one of the co-primary endpoints, had a 10.6 months median survival in the bevacizumab arm compared to median survival of 6.2 months for standard therapy with a hazard ratio (HR) of 0.64 (95% CI: 0.55–0.74, *p <* 0.0001). The central Independent Review Facility Assessed PFS (a secondary endpoint) demonstrated a median survival in the bevacizumab group as 8.4 months compared with 4.3 months in the standard therapy group; again statistically significant with stratified HR of 0.61 (95% CI: 0.53–0.71, *p <* 0.0001*).* The interim OS analysis demonstrated a small non-statistically significant benefit in the bevacizumab group with a one year survival rate of 72% (68–76) compared with 66% (62–71) with *p* = 0.052; with 254 events in the bevacizumab arm compared with 263 events in the standard therapy arm, with a stratified HR of 0.89 (0.75–1.07), *p* = 0.2135. In the arm receiving up-front bevacizumab, there were statistically significant benefits in the five pre-specified domains of health-related quality of life (HRQoL) (secondary endpoints), namely global health status, physical functioning, social functioning, motor functioning, and communication deficit; with longer median duration that patients were stable/improved from baseline. The median duration these patients maintained was a KPS higher than 70, with 9 months in the bevacizumab arm *vs*. 6 months in the standard arm. In the bevacizumab arm, 66% of patients who were on steroids at baseline discontinued their steroids compared with 47% in the standard arm. The time to steroid initiation for patients who had been off steroids at baseline was 12.3 months in the bevacizumab arm *versus* 3.7 months in the standard arm with a HR of 0.71 (95% CI;0.57–0.88, *p =* 0.0018*).* Overall, patients receiving bevacizumab had a diminished steroid requirement. There was no significant increase in intracranial hemorrhage (2.6% *vs*. 2.2% for all WHO tumor grades, I–IV, 1.5% *vs*. 0.7% for grade 3 or higher). There was more mucocutaneous bleeding in the bevacizumab arm (26.7% *vs*. 8.9%), but only 0.4% was grade 3 or higher. There were slightly more wound-healing complications (3.7% *vs*. 2.2%), as well as an increase in arterial thromboembolic events (5% *vs*. 1.6%) and a slightly lower incidence of venous thromboembolic events (7.8% *vs*. 9.6%). The incidence of hypertension was higher in the bevacizumab arm; 37.5% *vs*. 13.0%, and 10.3% *vs*. 2.0% for grade 3 or higher). Proteinuria was also higher (14% *vs*. 4%). There was a slight excess of gastrointestinal perforation at 1.7% *vs*. 0.2%; with abscesses or fistulae of 0.6% *vs*. 0.4%.

The other phase III study, RTOG 0825, also reported a longer PFS in the bevacizumab arm compared to the standard therapy; 10.7 months, and 7.3 months, respectively, HR = 0.79 (95% CI; 0.66, 0.94, *p* = 0.007), which actually did not reach the pre-specified statistical endpoint of *p* = 0.004. The mOS could not achieve the pre-specified statistical endpoint of *p* = 0.046, as that in the bevacizumab arm was 15.7 months and that in the placebo arm was 16.1 months (HR = 1.13, 95% CI; 0.93, 1.37). One of the striking differences between the two phase III trials was in the analyses of HRQoL data. In the RTOG 0825 trial, patients on the bevacizumab arm experienced significant worsening in cognitive function, motor dysfunction, and communication deficits measured by EORTC/BN20 QoL scale. Global symptom burden, interference and multiple factor groups measured by MDASI-BT were also significantly worse with bevacizumab compared to placebo. If longer PFS but equal OS compared to placebo, rather worse HRQoL (if the analyses of HRQoL for RTOG 0825 was appropriate), and some side effects are realistic, it would suggest no room for bevacizumab to be used in an upfront GBM treatment setting. 

## 4. Treatment Options after Progression on Bevacizumab

It is also challenging to determine treatment options for patients with recurrent GBM who progress after bevacizumab treatment. Currently there is no active regimen in this setting, since previous studies that employed continuing bevacizumab plus another agent, for example irinotecan, carboplatin, etoposide, or dose-intensified TMZ, or discontinuing bevacizumab and treating with an alternative agent failed to show efficacy: mPFS was 1.0–2.6 months, PFS-6m was 0–16%, and mOS was 3–6 months [9,25,26,27].

There is a concern that discontinuation of bevacizumab after failure in patients with recurrent GBM, may give rise to rapid tumor re-growth with accelerated clinical deterioration, which is recognized as a rebound phenomenon [28]. Mikkelson and his colleagues reported that 28% of patients who did not respond to bevacizumab showed rebound progression and mOS was only 7 weeks [28]. Some of these patients exhibited a partial response to re-administration of bevacizumab post rebound progressive disease (PD). It is, however, argued that the incidence of rebound phenomenon after bevacizumab discontinuance is relatively rare in other studies, and it may be a matter of interpretation as to the definition [29,30,31].

Another critical issue that has been addressed is whether continuation of bevacizumab beyond progression (BBP) could enhance survival of patients with recurrent GBM, although its net benefit appears limited as described above. This therapeutic approach has been exploited and proved meaningful in metastatic colorectal cancer in ML18147 randomized phase III study [32]. Patients who received bevacizumab plus standard first line chemotherapy (either oxaliplatin or irinotecan) and exhibited PD were randomly allocated to standard second line chemotherapy either with or without bevacizumab until PD. OS from randomization, the primary endpoint, was significantly longer in patients with bevacizumab continuation than those without, with HR = 0.81 and *p* = 0.0062. PFS was also longer in the bevacizumab group (HR = 0.68, *p* < 0.0001) [32]. According to the positive results in colorectal cancer, and the fact that outcome after bevacizumab failure remains dismal, BBP has also drawn attention for recurrent GBM [22,25]. Reardon *et al*. analyzed outcome among patients with recurrent GBM who received subsequent therapy after initial progression on bevacizumab regimens of one of five consecutive, single-arm phase II trials; the bevacizumab partners were either irinotecan, daily TMZ, etoposide, bortezomib, or erlotinib [33]. mOS and OS at 6 months for patients who continued bevacizumab therapy beyond progression (*n* = 55) were 5.9 months and 49.2%, compared with 4.0 months and 29.5% for those treated with a non-bevacizumab regimen (*n* = 44; *p =* 0.014), and bevacizumab continuation was an independent predictor of improved OS (HR = 0.64; *p* = 0.04) [33]. OS for patients who did not receive further therapy after initial PD was only 1.5 months. To confirm whether BBP strategy is beneficial in recurrent GBM, a phase IIIb randomized trial (MO28347) has been planned to initiate in 2013 with estimated enrollment of 510 patients (NCT01860638). Patients with newly diagnosed GBM who are treated with standard TMZ and radiotherapy plus upfront bevacizumab will be randomized upon PD to receive second line therapy either with bevacizumab continuation *vs*. placebo, which will further proceed to the third line therapy. The primary endpoint is OS from randomization. 

## 5. Should Bevacizumab Be Used at First Indication of PD?

Another issue in treating recurrent GBM with bevacizumab that has not been treated with bevacizumab upfront is whether to apply it at the first relapse or to defer until other second line therapies have failed. As discussed above, despite its potent and rapid antitumor or anti-edema effects, tumors will eventually regrow and, at the time of bevacizumab failure, survival expectation is quite limited because of lack of effective follow-up therapies [9,25,26,33]. In the BRAIN trial (bevacizumab *vs*. bevacizumab + irinotecan), 85 patients in the bevacizumab alone arm comprised 69 (81%) at the first relapse and 16 (19%) at the second. mOS after initiation of bevacizumab was similar in both settings, 9.1 months and 9.2 months, respectively [7]. Omuro *et al*. performed a clinical trial of protracted TMZ regimen in patients with recurrent TMZ-pretreated GBM with or without a history of bevacizumab use. Patients with previous bevacizumab exposure (18 cases) survived significantly less than bevacizumab-naïve patients (19 cases; nine of them received bevacizumab after progression on protracted TMZ) (mOS was 4.3 months *vs*. 13 months, HR = 3.2; *p =* 0.001) [34], suggesting that bevacizumab may benefit patients with recurrent GBM given even at the second relapse. Piccioni *et al*. analyzed retrospectively patients with recurrent GBM treated with bevacizumab (388 cases) to determine whether the timing of bevacizumab initiation would affect time to progression (TTP) and post-bevacizumab survival after initiation of bevacizumab. Analysis of this data showed that there were no significant differences in any survival periods as median TTP was 4.1, 4.2, and 3.4 months for those treated with bevacizumab at the first recurrence (*n* = 264), at the second recurrence (*n* = 88), and at the third recurrence (*n* = 36), respectively (*p =* 0.165), and median post- bevacizumab survival was 8.5, 8.9, and 6.2 months, respectively (*p =* 0.330) [35]. Since these studies are not intended to evaluate this issue primarily, efficacy and its duration of chemotherapy may be reduced with an increase in number of recurrence. Prospective studies are warranted to address this question. Nonetheless, these results may pose a potential strategy to withhold bevacizumab until further recurrence occurs by applying novel exploratory therapeutics in front at the initial recurrence prior to bevacizumab to benefit survival in cases with good performance status. 

## 6. Recurrence with Diffuse Infiltrative Pattern after Bevacizumab Therapy

It has been postulated that anti-angiogenic therapy, such as bevacizumab treatment of GBM, results in an increased incidence of distant and diffuse patterns of radiographic recurrence. One of the potential explanations for this phenomenon is that antiangiogenic therapy only targets the angiogenic-dependent contrast-enhancing components of tumor, but does not target the angiogenic-independent, highly-infiltrating glioma cells at the leading edge demonstrated by FLAIR image [29,36]. Another reason is that after normalization of VEGF-induced abnormally developed tumor vasculature and interstitial edema, remaining glioma cells migrate through preexisting vessels by vessel cooption [37]. Iwamoto *et al*. reported on patterns of relapse in 37 adult patients with recurrent GBM treated with bevacizumab [38]. Following progression on therapy, 35% of patients showed non-enhancing tumor progression. Norden *et al*. retrospectively evaluated 28 patients with high-grade gliomas treated with bevacizumab and irinotecan with respect to the pattern of disease progression after bevacizumab failure; 18% were diffuse and 18% were distant. However, they also found similar patterns of radiographic recurrence (18% diffuse and 6% distant) in those not treated with bevacizumab [27,39]. Pope *et al.* retrospectively analyzed the data of the prospective BRAIN trial of 167 patients with recurrent GBM treated with bevacizumab and with or without Irinotecan. There were 17% diffuse and 2.4% distant patterns of recurrence after the Stupp regimen prior to bevacizumab. Following bevacizumab treatment, 82% of patients maintained the same in disease pattern, and 16% of patients in the baseline local disease group were converted to a diffuse pattern [40]. Chamberlain *et al*. retrospectively reviewed 80 patients with GBM who were treated with the Stupp regimen initially, followed by bevacizumab monotherapy at first recurrence as for patterns of radiographic presentation. At initial diagnosis, 87.5% of GBM were local and 2.5% were diffuse. At first recurrence after the Stupp regimen, 80% were local and 6.25% were diffuse. At second recurrence following progression on bevacizumab, 71% were local and 11.25% were diffuse, and at third recurrence, they were 71% and 14%, respectively, suggesting that the majority of patients with GBM manifest local disease and maintain the same pattern regardless of multiple recurrence and use of bevacizumab [41]. This issue has been addressed in a prospective manner in a large international phase III AVAglio trial for patients with newly diagnosed GBM as discussed earlier. Patterns of radiographic progression were assessed in 65% of patients enrolled in the study as compared to the baseline. At baseline, a diffuse pattern was observed in 60% of placebo arm and 70% of bevacizumab arm. Non-diffuse tumors (placebo arm 40%; Bev arm 30%) changed to a diffuse pattern at progression in 22.8% and 24.7% cases, respectively, indicating no significant increase in induction of an invasive pattern of recurrence following upfront bevacizumab treatment [42]. Based on these data from clinical trials and experiences, it does not seem a universal phenomenon that anti-angiogenic therapies induce preferentially diffuse invasive progression in GBM, although a number of basic research studies have implicated it through several distinct mechanisms including a shift of major pro-angiogenic factors from VEGF to others, such as c-Met or SDF (discussed later), and it is still challenging to define response and progression radiographically following bevacizumab treatment which may result in different interpretation of patterns of recurrence. 

## 7. Bevacizumab Dosing

As bevacizumab is usually administered at 10 mg/kg, every 2-week interval, it is still unclear which dosing schedule is the most optimal for bevacizumab monotherapy in recurrent GBM. To analyze its dose-response effect, Wong *et al*. performed a meta-analysis of 15 studies published from 2005 to 2009, involving 548 patients treated with bevacizumab for recurrent GBM and showed that there was no difference in bevacizumab dose-response benefit between 5 mg/kg and 10 to 15 mg/kg [43]. If the dosing could be lowered to 5 mg/kg, reduced bevacizumab use results in a substantial savings of medical cost. The cost-effectiveness of 5 *vs*. 10 mg/kg of bevacizumab dose in GBM patients needs to be prospectively examined. 

## 8. Next Steps and Future Directions

Disease progression, as reflected by tumor growth and, probably enhanced, metastasis/invasion during treatment with inhibitors of VEGF signaling, is attributed to multiple interacting mechanisms. Among them are activation of pathways that favor epithelial-mesenchymal transition, promotion of invasiveness, and induction of tolerance and activation of cancer stem cells. Disease progression during treatment with bevacizumab paired with chemotherapy does not necessarily mean that the inhibitor has lost efficacy [44]. The resistance could be due to the chemotherapy. Evidence of better OS in metastatic colorectal cancer, when bevacizumab is continued beyond progression in the presence of diverse types of chemotherapy, reflects the continued involvement of VEGF as mentioned before [45].

However, some effects of VEGF inhibitors that slow tumor growth can still promote invasion and metastasis in certain models [46,47]. Tumors with high MET expression or activating mutations of MET are generally more aggressive and have a less favorable prognosis [48]. The activation of MET can promote epithelial-mesenchymal transition and tumor invasiveness [49,50], partly by increasing the activity of transcriptional repressors, such as snail homolog 1 (*SNAI1*; also known as *SNAIL*), *ZEB1* and *TWIST1*, that reduce E-cadherin expression, increase N-cadherin expression and turn on the expression of other mesenchymal markers [51]*.* Inhibition of VEGF signaling can result in decreased expression of epithelial markers and increased expression of mesenchymal markers such as SNAI1, TWIST1 in preclinical models [52,53]. The expression of the mesenchymal marker fascin increases in GBMs after treatment with bevacizumab [54].

Targeting angiogenesis and tumor progression/invasion/metastases together has recently shown promise as a strategy for preventing escape from inhibitors of VEGF signaling [54]. One approach is the inhibition of MET and VEGF signaling together, either by concurrent administration of selective inhibitors or by single agents that block both receptors [cabozantinib (also known as XL184) or foretinib (also known as XL880)] [55,56]. Concurrent inhibition of MET and VEGF signaling can slow tumor growth, decrease invasion and metastasis, and change invasive tumors into a shape with a ball-like appearance in certain models. The therapeutic benefit of this approach is currently being evaluated in clinical trials of multiple tumor types. 

Cabozantinib, which inhibits MET, AXL and VEGF receptors, as well as multiple other receptor tyrosine kinases, is a potent inhibitor of invasion and metastasis in spontaneous and xenograft tumors in mice [55,56]. Cabozantinib has better effects on tumor angiogenesis and survival than those found with combinations of selective inhibitors of MET and VEGF signaling in the same model, suggesting that AXL or other targets (such as RET, KIT and TIE2) contribute to the efficacy of cabozantinib [57]. Cabozantinib is showing promising results in clinical trials of castration-resistant metastatic prostate cancer, medullary thyroid cancer, breast cancer, NSCLC, malignant melanoma, liver and ovarian cancers [58,59]. It remains to be determined if clinical trials with such dual or multiple inhibitors will effectively impact GBM recurrence and invasion. Blocking both angiogenesis and escape pathways is now an attainable step in the evolution of the use of agents that inhibit VEGF signaling together with other targets. The current knowledge of tumor vascular biology and mechanisms of tumor growth, invasion and metastasis, will enable these approaches. Steps that still need to be taken include learning more about escape mechanisms, identifying additional targeted drugs that act synergistically with angiogenesis inhibitors. Predictive biomarkers to identify patients who will have benefit from such approaches is also important. 
